# Supplementary material for: Caregiver transformation and relational growth in a parent-mediated intervention for autism in Hong Kong – A qualitative study
Source: PLOS Ment Health. 2025 Oct 24;2(10):e0000263. doi: 10.1371/journal.pmen.0000263 (PMC12798404; doi:10.1371/journal.pmen.0000263)
Supplement: S1 Table — (DOCX) [file pmen.0000263.s002.docx]

**Parent-child relationship – Supporting Information**

**S1 Table**

**Focus group characteristics**

| Group | Relationship with child | Fieldwork location | Moderators |
| --- | --- | --- | --- |
| 01 | Mother x1  Aunt x1 | The University of Hong Kong | Main: Second author  Assistant(s): First author |
| 02 | Mother x7 | Online via ZOOM | Main: Second author  Assistant(s): First author |
| 03 | Mother x4 | Online via ZOOM | Main: First author  Assistant(s): Second author |
| 04 | Mother x4  Grandmother x1 | Partner NGO’s centre | Main: Second author  Assistant(s): First and third authors |
| 05 | Mother x3  Father x1 | Online via ZOOM | Main: Second author  Assistant(s): NA |
