# Supplementary material for: Caregiver transformation and relational growth in a parent-mediated intervention for autism in Hong Kong – A qualitative study
Source: PLOS Ment Health. 2025 Oct 24;2(10):e0000263. doi: 10.1371/journal.pmen.0000263 (PMC12798404; doi:10.1371/journal.pmen.0000263)
Supplement: S2 Table — (DOCX) [file pmen.0000263.s003.docx]

**Parent-child relationship – Supporting Information**

**S2 Table**

**Participants characteristics**

| Participants information | No. of participants |
| --- | --- |
| **Age** |  |
| 20-29 | 1 |
| 30-39 | 15 |
| 40-49 | 5 |
| 50-59 | 1 |
| **Marital status** |  |
| Single | 3 |
| Married | 16 |
| Separated or divorced | 3 |
| **Education levels** |  |
| Secondary school | 9 |
| Post-secondary diploma | 4 |
| Bachelor | 7 |
| Master or above | 2 |
| **Work status** |  |
| Work full time | 5 |
| Work part time or multiple jobs | 3 |
| Homemaker | 14 |
| **Household income** |  |
| HK$19,999 or less | 3 |
| HK$20,000 to HK$29,999 | 5 |
| HK$30,000 to HK$39,999 | 3 |
| HK$40,000 to HK$49,999 | 2 |
| HK$50,000 to HK$79,999 | 5 |
| HK$80,000 or above | 1 |
| Refuse to answer | 3 |
| **Living space (per person)** |  |
| ~4.65 square metre or less  　(50 square feet or less) | 2 |
| ~4.66 - ~9.29 square metres 　(51-100 square feet) | 8 |
| ~9.30 - ~13.94 square metres 　(101-150 square feet) | 6 |
| ~13.95 - ~18.58 square metres 　(151-200 square feet) | 4 |
| ~18.60 square metres or above 　(201 square feet or above) | 1 |
| Children’s information |  |
| **Age of autistic child** |  |
| Less than 4 years old | 4 |
| 4 to less than 5 years old | 10 |
| 5 to less than 7 years old | 5 |
| 7 years old or more | 3 |
| **Year of children being diagnosed** |  |
| 2 years or less | 10 |
| More than 2 years to 3 years | 7 |
| More than 3 years to 4 years | 3 |
| More than 4 years | 2 |
| **Any siblings** |  |
| Yes, with developmental delay | 5 |
| Yes, without developmental delay | 4 |
| No siblings | 13 |
