# Supplementary material for: Caregiver transformation and relational growth in a parent-mediated intervention for autism in Hong Kong – A qualitative study
Source: PLOS Ment Health. 2025 Oct 24;2(10):e0000263. doi: 10.1371/journal.pmen.0000263 (PMC12798404; doi:10.1371/journal.pmen.0000263)
Supplement: S3 Table — (DOCX) [file pmen.0000263.s004.docx]

**Parent-child relationship – Supporting Information**

**S3 Table**

**Themes and sub-themes relating to parent-child interaction**

| Theme 1 | Caregivers’ paradigm shift: From care providers to persons with growth |
| --- | --- |
| *Sub-themes* | 1.1 From knowledge-based to practice-based   - 1. From technical to authentic engagement |
| Theme 2 | Changes of children’s status: From care recipients to stakeholders |
| *Sub-themes* | 2.1 Fostering fluid and connected relationships  2.2 Parent-child togetherness |
