# Supplementary material for: Caregiver transformation and relational growth in a parent-mediated intervention for autism in Hong Kong – A qualitative study
Source: PLOS Ment Health. 2025 Oct 24;2(10):e0000263. doi: 10.1371/journal.pmen.0000263 (PMC12798404; doi:10.1371/journal.pmen.0000263)
Supplement: S1 Text — (DOCX) [file pmen.0000263.s005.docx]

**Parent-child relationship – Supporting Information**

**S1 Text**

**Faculty of Social Sciences, The University of Hong Kong**

**Guide for Focus Groups with Caregiver Training Participants**

This focus group guide targets at the participants of the caregiver training. It is designed to solicit their experiences of and perceptions on the impacts of the program on their lives and the people around them.

**Introduction**

1. How old is your kid? When was s/he diagnosed with ASD?
2. What motivated you to participate in this course?

**Impact Mapping**

1. What did you get from this course?
2. Do you notice any changes in you, your child or the people around you because of this course? What are they?

**Social sustainability**

1. What makes it easy or difficult to apply the knowledge and skills?
