# Supplementary material for: Caregiver transformation and relational growth in a parent-mediated intervention for autism in Hong Kong – A qualitative study
Source: PLOS Ment Health. 2025 Oct 24;2(10):e0000263. doi: 10.1371/journal.pmen.0000263 (PMC12798404; doi:10.1371/journal.pmen.0000263)
Supplement: Attachment — Submitted filename: Response to reviewers v2.0.docx [file pmen.0000263.s007.docx]

Dear Editor and Reviewers,

We sincerely wish to thank you for your inspiring comments. Please find below our point-to-point comments.

**Editor: Which language were the discussions with parents conducted in?**

**Response**: Thank you for the question. The focus group discussions were conducted in Cantonese and transcribed into traditional Chinese. We have added this information in lines 204-206 in track-changes version (lines 132-134 in the clean version). We also added that the quotations used were translated into English during manuscript preparation and that the translation was approved by all authors (lines 236-237 in track-changes version and lines 164-165 in the clean version).

**Editor:** Additional details could be provided regarding "hermeneutic constructivism" and "interpretivist epistemology". The former is described in the current manuscript as acknowledging that "reality is socially constructed". Some attention should be given to this description, which seems to endorse a view that objective truth does not exist -- is this appropriate for a scientific journal?

**Response**: We sincerely appreciate the editor for raising this thoughtful point. We recognise the importance of clearly articulating these philosophical foundations and have updated the “study design” section in lines 174-189 in track-changes version (lines 105-115 in the clean version).

Hermeneutic constructivism emphasises that human understanding of reality is inherently shaped by individual perceptions, cultural contexts, and social interactions, but without rejecting the existence of objective truth.

This perspective aligns with our research as we explored and interpreted participants’ subjective meanings of parent-child relationship post-intervention, which helps uncovering their ideas and meaning of relationships rather than attempting to establish universal truths.

In terms of alignment with scientific rigor, we sincerely believe that constructivist approaches are widely accepted in qualitative research, particularly in fields like psychology, social work, education, and health sciences, where understanding lived experiences is critical for informing practices and policies.

**Reviewer 1:** The authors' approach to including a thematic analysis is valuable given the need for more qualitative work/lived experience in the context of psychopathology. I am not an expert in qualitative analyses so I would defer to the expertise of other reviewers in terms of the methodological rigor of the approach taken.

**Response**: Thank you for the comment.

**Reviewer 1:** However, the limited novelty in the specific questions leveraged coupled with other concerns (small sample size, limited generalizability to different family members, a lack of a counterfactual to compare differences between those with and without intervention, transferability of the current findings to other contexts, etc.) made me waver on the added value of publishing this work over others.

**Response**: Thank you for pointing out this point.

Our study aimed to collect the lived experiences of people who participated in the programme without aiming to generalize to the majority who may like to join the programme. Yet, we had reported the demographics of the participants and wished that readers and potential service users may decide about the implications of the findings. The diversity of participants (including mothers, fathers, aunts, and grandmothers; see lines 248-250 in track changes version or 176-178 in clean version) allowed us to capture the unique experiences of caregivers in different roles, enriching the depth and breadth of the discussion.

We also added that future research could benefit from incorporating a counterfactual comparison in lines 623-625 in track-changes version (lines 461-463 in the clean version).

Thank you for raising the transferability concerns of our findings. Although this research focuses on caregivers of autistic children, the identified themes and strategies may also benefit parents of other neurotypical children and inform boarder family dynamics. We have updated lines 526-531; 536-545; 567-569 in the track changes version (lines 396-400; 402-408; 423-426 in the clean version), suggesting the core parenting principles this intervention offer may benefit parents of neurotypical.

**Reviewer 2:** The article presents a well-structured and well-written qualitative study into the experiences of caregivers of Autistic children. Changes in caregivers’ perception of their relationship with the child after a parent-medicated intervention are discussed within the cultural context. The study documents first-person accounts of what works for promoting a positive and inclusive environment within the family of Autistic children, surrounded by a less inclusive society that values achievements and compliance with social norms. These accounts deserve to be heard and published, and the paper contributes to this goal in a rigorous scientific manner.

**Response**: Thank you.

**Reviewer 2:** To improve the paper, I suggest enhancing the discussion that currently remains very close to the study findings and would benefit from a broader reach into the research and social context. This can be done by comparing the findings with other studies of parent-mediated or relation-focused interventions, including from different cultures. Another way of enriching the discussion is by discussing the study within the neurodiversity-affirmative movement, relevant to the study focus.

**Response**: We very much appreciate your suggestions and direction.

We have added literature that compares our findings with existing parent-mediated intervention (lines 457-460 in track changes and 374-377 in clean version); its potential application to neurotypical population (lines 526-531, 543-545, 567-569 in track changes and 396-400, 405-408, and 423-426 in clean version)

**Reviewer:** The paper itself showcases the difficulties of navigating the social context while valuing an Autistic child’s needs and agency. It expresses a neurodiversity-affirmative mindset using a deficit-based language (‘with ASD’) that is compliant with the professional social norms set by the DSM. It is focused on a family intervention empowering the child but views the goal of this intervention as that ‘improved ASD child behaviour, facilitated by parent-mediated interventions, supports smoother integration into societal norms, ultimately promoting social inclusion.’ This perspective links inclusion to the child's compliance with societal expectations, while in Western cultures, inclusion is viewed as the societal environment accommodating diverse ways of being.

**Response**: Thank you for bringing this very important point to our attention.

We agree the necessities of adopting neurodiversity-affirmative language and revised the whole manuscript accordingly. We had to maintain the DSM terminology in the introduction of the manuscript for clarity for a traditional readership but reworked rather heavily the rest of the manuscript with more inclusive and strengths-based language among the all authors also shared the recent view.

We also appreciate your observation regarding the intervention’s framing, which may give the impression in linking social inclusion to the child’s adherence with societal norms. We agree that in many Eastern cultures like Hong Kong, societal harmony and group cohesion can unintentionally shape approaches to inclusion. We have clarified in the manuscript that while positive behavioural outcomes may facilitate smoother interactions within societal contexts, the focus of the intervention is on fostering mutual understanding, acceptance, and respect within family units (lines 121-127 for track-changes version, and lines 87-91 in the clean version). We also added in the conclusion that inclusive and context-sensitivity strategies can empower family and honours neurodivergent identities (lines 650-655for track-changes version, and lines 479-481 in clean version).

**Reviewer:** Not surprisingly, the text, challenging cultural norms while complying with them, reflects the cultural conflict addressed by the studied intervention, as well as the conflict between the behavioural philosophy of medicine and the person-centred philosophy of qualitative research. These controversies, affecting not only Autistic individuals and their families but also professionals supporting and researching them, deserve to be addressed in an open and reflective manner.

**Response**: Thank you so much for this comment very sincerely.

We appreciate your recognition of the inherent tension between challenging dominant cultural narratives and operating within them, as well as the broader philosophical contrast between behavioural models in clinical practice and person-centred approaches in qualitative research. We have added a paragraph in the discussion (lines 572-585 in track changes and lines 428-441 in clean version) to acknowledge these complexities.

**Reviewer:** Did any part of the text analysis use natural language processing? Why or why not?

**Response**: Thank you for your question.

No part of the text analysis has used natural language processing. Instead, all coding and analysis were conducted manually, supported by NVivo software for data organization (lines 228-231 in track changes and lines 156-159). This decision was intentional, as we believe natural language processing lacked the direct engagement with the data involving the complexity of participants’ lived experiences and may not be the best idea to capture nuanced meanings and contextual subtleties needed for this interpretive qualitative analysis.

Additionally, since all focus group discussions were conducted in Cantonese (a spoken form of one of the many Chinese languages). Unlike standard written forms of Chinese, Cantonese has rich contextual and cultural nuances that are challenging to interpret or process. Current automated natural language processing tools which are often optimized for written Chinese, are less effective at capturing the underlying meanings and contextual subtleties of spoken Cantonese.

(Reference: Xiang, R., Chersoni, E., Li, Y. *et al.* Cantonese natural language processing in the transformers era: a survey and current challenges. *Lang Resources & Evaluation* (2024). https://doi.org/10.1007/s10579-024-09744-w).

For these reasons, we opted for manual coding which allowed the researchers to remain reflexive, iteratively revisiting themes and critically assessing their interpretations to ensure the findings were grounded in participants’ voices.

**Reviewer:** Please include more details regarding the parent-mediated interventions and the caregiver training.

**Response**: Thank you. We have added details regarding the intervention, in lines 98-111 of the track changes version and lines 73-82 in the clean version.

**Reviewer:** Please verify whether the deidentified text transcripts can be included to satisfy the PLOS Data Policy. If data sharing is permitted, then public archiving is preferred over "reasonable request" gatekeeping.

**Reply**: Thank you for raising this point. Although we also believe in data transparency, due to the potential violations of the sought consents from participants and funder, we are more than happy to share the deidentified text transcripts written in the language being used in the focus group discussions by request.

**Reviewer:** Where feet are used as units, please also provide an equivalent measurements in meters.

**Response**: Thank you. We have converted all units and kept the feet in parenthesis.

Your valuable insights have greatly contributed to improving the manuscript. We look forward to your further feedback.

Regards,
Camille Chan

On behalf of all co-authors
