# Supplementary material for: Caregiver transformation and relational growth in a parent-mediated intervention for autism in Hong Kong – A qualitative study
Source: PLOS Ment Health. 2025 Oct 24;2(10):e0000263. doi: 10.1371/journal.pmen.0000263 (PMC12798404; doi:10.1371/journal.pmen.0000263)
Supplement: Attachment — Submitted filename: Response_to_reviewer_v3.docx [file pmen.0000263.s008.docx]

Dear Reviewer 3,

We sincerely thank you for your thoughtful comments, which have been invaluable in helping us improve the manuscript. Below, we provide our point-by-point responses to your feedback

Reviewer #3: This paper addresses an important topic – caregiver experiences of a particular parent-mediated therapy – within a particular cultural context. Parent-mediated therapies have an emerging body of evidence in the autism field, albeit most research has focused on child-related quantitative outcomes (as acknowledged by the authors).

There are some aspects to the paper that I feel would need addressing/resolving before this paper would be suitable for publication.

**Response**: Thank you. We appreciate your insights and your recognition of the merits of our work.

Reviewer #3: One aspect that I think needs more consideration from the authors is addressing the tension in the paper of referring to working within ‘neurodiversity-affirming approaches and frameworks’ while at the same time, some of the language used in the paper (e.g., “ASD children” – more preferred language amongst the autistic and autism communities is ‘autistic children’) and some of the approaches and outcomes reported that focus on child-related behaviours that were targeted (e.g., lines 263-269 of the clean version, starting at “A mother shared how her child would throw toys during tantrums….”) would not align with this approach. I note that there may be cultural differences in what is considered ‘neurodiversity-affirming’ and that I may be bringing a Western conceptualisation, however, I think it would be important for the authors to address this point if that is the case.

**Response**:

Thank you for your observations and comments. The concept of “neurodiversity” has been advocated in Hong Kong and yet, this concept is still very noel to many in the general population. Hence, it is very common to see that there are still gaps between the languages in describing ASD between the service users and the professionals and researchers not only in the manuscript but also in the daily conversations.

While we previously included the concept of neurodiversity in the first paragraph of the Introduction, we have now moved it to the Discussion (lines 434-439). This allows us to, first, define ASD clinically while reserving the broader discussion of neurodiversity for later, in alignment with the themes explored in the study.

Nonetheless, we have revised several statements that are more aligned with the neurodivergent ideology; for instacnes, (previous) lines 263-269:

(Track changes) Lines 283-286:

A mother recounted how her child used to throw toys during moments of distress and described applying the facilitator’s advice to introduce gentle boundaries to help the child process these situations.

We have added a sentence to elaborate on our cultural observation regarding autistic individuals:

(Track changes) Lines 247-250:

While the term ‘misbehaving’ may not align with a neurodiversity-affirming perspective, its use by the caregiver reflects both a developing awareness of the child's underlying needs and emotional triggers, while also reflecting a common parental language in Hong Kong for unexpected behaviour.

(Track changes) Lines 439-441:

However, some caregivers have yet to adopt neurodiversity-affirming language, possibly influenced by cultural norms and limited familiarity with this perspective.

We also conducted a thorough review of the manuscript to ensure it aligns with a neurodiversity-affirming framework. The revised lines (highlighted in the updated track changes version) include the following:

- Lines 71-73: To address these needs, a culturally adapted parent-mediated intervention was introduced to support Hong Kong caregivers raising autistic children.
- Lines 117-119: …explored caregivers’ lived experiences after participating in a parent-mediated intervention designed to support caregivers of children diagnosed with, or suspected to be on, the autism spectrum.
- Lines 138-139: …who were experienced in working with autistic children and with healthcare providers, ensured rigorous study execution.
- Lines 200: … and the age of their autistic child, is presented in Table 1.
- Line 403: The study explored the lived experiences of caregivers of autistic children…
- Lines 565-568: These tensions not only shape the experiences of neurodivergent individuals and their families, but also challenges researchers and practitioners to adopt inclusive, reflective practices within complex sociocultural settings (47).

Reviewer #3: Without more *detailed information about the intervention (**e.g., size of the groups, length of sessions, who attends the sessions*, delivery mode, key strategies, theory of change, active ingredients etc) it is also difficult to ascertain if the entire therapy program itself would align with a neurodiversity affirming framework.

**Response**: Thank you. Regarding the alignment with the neurodiversity-affirming framework, the invention was co-designed by the WHO and Autism Speaks, both recognised experts in the field. Thus, we are confident that the localised program adhers to a neurodiversity affirming framework. To make this clearer, we have revised the introduction (lines 73-92) to indicate that the PMI discussed in the manuscript is the localised version of WHO-CST. We also clarified details about the session participants, number of sessions, key strategies, theory of change, and active ingredients. It revised text now reads:

“The intervention evaluated in this study was adapted from the World Health Organization Caregiver Skills Training (WHO-CST) program. This program is designed to improve the confidence, parenting skills, and overall well-being of caregivers raising children with developmental delays by using evidence-based parenting and parent-mediated intervention strategies (11). It was introduced locally in 2018 through the Jockey Club Autism Support Network and further localized in 2021 by the local NGO master trainers to reflect Hong Kong’s specific cultural and caregiving context (12). The programme teaches caregivers of children aged two to six with responsive caregiving strategies such as developing routines and introducing tools for emotional attunement and stress management. Across nine structured group-based sessions, caregivers are guided to integrate practical approaches into daily play and home activities, which becomes opportunities for child development. Rather than positioning the caregivers as therapists, the training empowers them to be responsive, confident, and emotionally attuned with their child’s unique developmental journey. Key objectives of the programme include strengthening caregiver-child interactions, supporting children’s learning and communication, enhancing caregiver well-being. These elements work together to shift caregivers’ perspectives—not only helping them manage challenges but also encouraging them to embrace their child’s identity and potential, forms the foundation of the programme explored in this study.”

We have added further information in the Materials and Methods section. These revisions include details about the program’s origin, group size, session length, and medium of delivery. The updated text (lines 131-134) states:

“The parent-mediated intervention examined in this study was a culturally adapted program based on the WHO-CST model. Further details of the intervention’s structure are published elsewhere (12, 24). Each group cohort comprised approximately six to eight caregivers, and the program consisted of nine structured sessions, each lasting between 90 and 120 minutes (25).”

We have also included the mode of delivery as a limitation in the manuscript (in lines 535–539) to acknowledge its potential influence on the outcomes.

“Second, depending on pandemic-related restrictions and service availability, some groups received the intervention through in-person classes with home visits, while others participated via Zoom and submitted video-based exercises for facilitator feedback (24). This may have influenced how caregivers experienced support and applied strategies at home.”

Reviewer #3: The other aspect to the paper that requires more consideration is around the interpretations and conclusions from the data. There are multiple claims in the paper that I feel are not directly supported by the data being reported on and these need to be more accurately represented for publication purposes. For example:
• After a caregiver quote, the authors conclude “These practices helped caregivers foster a more supportive and emotionally stable environment at home.” – are there any data (beyond caregiver quotes) to support this extension?

**Response**: Thank you for pointing this out. We have revised the sentence you mentioned (now lines 307-309) and it now reads:

“Through these practices, caregivers learned to understand their own and their child’s emotions, fostering co-regulation and modelling calmer responses during emotionally charged situations.”

We also reviewed the manuscript to ensure that other claims are appropriately supported by the data. The sentences below are modified to reflect more conservative interpretations:

- Lines 220-222: It represents a transformative journey for caregivers, as they reported learning from the intervention, integrating this knowledge, and applying it in their interaction with their child.
- Lines 226-229: Caregivers in all five focus groups described a transition in their approach, moving from a parent-centred parenting model to one that recognises and accommodates the unique needs of each child.
- Lines 238-240: These insights demonstrate the ongoing learning process where caregivers integrated new knowledge from the intervention into their daily interaction with their child.
- Lines 241-242: Caregivers also reported self-reflection and personal growth as positive outcomes of the intervention.
- Lines 320-326: …acquiring practical skills can lead to meaningful transformation in caregivers’ parenting perspectives. The narratives of the caregivers demonstrate the potential of self-reflections for a broader process of growth in caregivers’ roles, building their confidence and resilience in supporting their child’s development, thereby strengthening the parent-child relationships.
- Lines 329-332: … reflects a transformation wherein caregivers perceive their child not as a passive recipients of care but as active participants to their relationships. The caregivers explained that there could be a potential paradigm shift among themselves in understanding of their child’s identity, as they observed what they perceived to be increased responsiveness from their child, particularly in emotional expression, independence, and trust.
- Lines 345-346: Participants observed this shift in the child’s enhanced emotional expression and regulation, increased joint engagement, and greater compliance.
- Lines 347-349: Participants observed improvement in their child’s emotional expression and self-regulation, which is understood as a hallmark of a balanced and reciprocated parent-child relationship.
- Lines 358-360: Caregivers reported observing reduced dependency and increased willingness to communicate and interact with one another, suggesting the intervention promoted independence and social engagement.
- Lines 371-375: This sub-theme emphasises the importance of emotional communication, trust, and shared understanding, as participants experiences found these elements encourage active participation in daily routines and joint activities.
- Lines 413-415: Participants commonly reported a transformation in their parenting approaches, marked by a shift toward more empathetic, responsive, and child-centred practices.
- Lines 426-428: Beyond acquiring the taught skills in the programme, caregivers reported personal growth through mindfulness, self-care practices, and exercises in emotional regulation.
- Lines 445-460: …caregivers began to recognise and attend to their own emotional needs, shifting their perspective of caregiving from solely child-centric to a fluid and relational process of mutual growth. This shift contributed to more connected and flexible parent-child relationships a more consistent practices of positive parenting. These findings align with previous research highlighting the importance of self-reflection, emotional attunement, and compassionate caregiving (33-35). Furthermore, many caregivers described their children not as passive care recipients, but as active participants in family life. Oruir research indicated that parenting programs broadly enhance children’s social, emotional, and behavioural development across clinical and non-clinical populations (36), suggesting that such interventions may benefit families beyong the neurodivergent community by equipping caregivers with developmentally supportive strategies.

To further authenticate our findings, we have added relevant supporting evidence from our earlier research in the discussion section as listed below. While this additional context may not directly extend support to the same level as the original quote, it provides valuable supplementary information and strengthens the overall alignment of our conclusions with the data.

- Lines 419-422: This aligns with our earlier findings during the adaptation and pretesting phase of the program, which reported improvements in caregiving skills, emotional self-management, handling of challenging child behaviours, and the perceived value of home visits (12).

Reviewer #3: • “This shift marks the evolution of their relationship from one-directional to reciprocal” – again, are there any data to support this? I think it could also be asserted that the relationship was always reciprocal, however, caregivers are now reporting feeling more attuned/connected (if I am interpreting the caregiver quotes accurately).

**Response**: Thank you for pointing this out. We have reviewed and revised the sentence (now lines 338-340):

“This shift appeared to correspond with caregivers’ perception of a more reciprocal and connected relationship as reflected in positive changes in both behaviour and parent-child interactions.”

Reviewer #3: • “Overall, this theme illustrated the cascading effects of caregiver transformation on children. As caregivers became more responsive and attuned to their child’s needs, children demonstrated greater emotional expression, independence, and social engagement. This reciprocal growth highlights how the intervention positively influenced both individual and relational dynamics, ultimately fostering a deeper, more collaborative parent-child relationship.” Without a mediation/mechanism analysis to support this claim, this should only be presented as a hypothesised pathway. I do not think this statement is supported by the available data).

**Response**: We appreciate your feedback. We have reframed the statement to clearly present it as a hypothesized pathway. The revised text now states (now lines 394-400):

“Overall, this theme illustrated the hypothesized cascading effects of caregiver transformation on children. In particular, how caregivers responsiveness and attunement were perceived to be associated with changes in their child’s needs in terms of emotional expression, independence, and social engagement. This perceived reciprocal growth highlights a potential pathway through which their newly adopted parenting approach may have positively influenced both individual and relational dynamics, fostering a deeper and more collaborative parent-child relationship.”

Reviewer #3: • “Our findings revealed a dual transformation: caregivers experienced a paradigm shift from task-focused care providers to reflective individuals engaging in personal growth, while children’s roles evolved from passive recipients to relational stakeholders within their families.” – without consulting children about their experiences (and without other child-outcome data), I feel this is an interpretation that is not supported by the direct data.

**Response**:

Thank you for pointing out the limitations of this claim. We have revised the statement to explicitly highlight that this interpretation is based on caregivers’ narratives. The revised text (lines 405-412) now reads:

“Our findings highlight a dual transformation as described through participants’ narratives. First, caregivers reported a shift from task-focused roles to more reflective, growth-oriented approaches, capturing their enhanced emotional regulation, self-care, and interactional skills. Second, caregivers perceived children as increasingly engaging as relational stakeholders within their families, with descriptions of children showing stronger emotional regulation, social engagement, and reciprocal communication. Together, these themes reflect a deepening synergy in the parent-child relationship as perceived by participants and shaped by the intervention.”

Additionally, we clarified that the qualitative nature of our study does not aim to produce generalizable results and acknowledged the absence of data from children’s perspectives (lines 532–535) as limitations.

“The voluntary nature of caregivers’ participation may have led to the inclusion of caregivers who had more positive experiences with the program, as well as not having evaluated changes from the child’s perspective.”

Reviewer #3: I acknowledge that by tempering these conclusions, it does further limit some of the outcomes/conclusions of the paper.

Finally, while I more than understood the paper, it would benefit from a detailed grammatical review by the authors to address the grammatical errors throughout.

**Response**: Thank you.

We have conducted a detailed grammatical review of the manuscript to address any remaining errors. We believe this process has contributed to a clearer and more polished version of the text.

We sincerely appreciate your thoughtful input. Your comments have been valuable in improving our manuscript.

Regards,
Camille Chan

On behalf of all co-authors
