# Supplementary material for: Caregiver transformation and relational growth in a parent-mediated intervention for autism in Hong Kong – A qualitative study
Source: PLOS Ment Health. 2025 Oct 24;2(10):e0000263. doi: 10.1371/journal.pmen.0000263 (PMC12798404; doi:10.1371/journal.pmen.0000263)
Supplement: Attachment — Submitted filename: Response_to_reviewer_v4.docx [file pmen.0000263.s009.docx]

Dear Reviewer 3,

Thank you for pointing out the sentences with grammatical errors. The sentences have been revised as follows:

Reviewer #3:
Line 33-34: “…context-sensitive strategies for environment with spatial limitations.” – sentence is unclear

**Response**: Thank you. We have revised the sentence to: “…context-sensitive strategies for small living environment.”

Reviewer #3:
Line 39: “…interventions can foster transformative in caregiver change”

**Response**: Thank you. We have revised the sentence to: “…interventions can encourage caregiver transformation…”

Reviewer #3:
Lines 77-80: “These elements work together to shift caregivers’ perspectives—not only helping them manage challenges but also encouraging them to embrace their child’s identity and potential, forms the foundation of the programme explored in this study.” – sentence is unclear

**Response**: Thank you. We have revised the sentence to: “The programme explored in this study is grounded in the concept that these elements work together to reshape caregivers’ perspectives, thereby enabling them to not only navigate challenges but also to foster their child’s identity and potential.”

Reviewer #3:

Lines 389-390: “..particularly for caregivers who are primary figures a child’s developmental journey.” Sentence missing the word “in”

**Response**: Thank you. We have added back the “in” and the sentence now reads: “…particularly for caregivers who are primary figures in a child’s developmental journey.”

Reviewer #3:

Line 395: “…with findings from internation studies” – should this be ‘international”?

**Response**: Thank you for picking up the typo. It has been revised to “international”.

Reviewer #3:

Line 416: “..Oruir research indicated…” – should be “Our”

**Response**: Thank you for picking up the typo. It has been revised to “Our”.

Reviewer #3:

418: “beyong” – should be ‘beyond’?

**Response**: Thank you for picking up the typo. It has been revised to “beyond”.

Reviewer #3:

494: “Consistent with neurodiversity-affirmative frameworks, these approaches shift the focus from behavioural normalisation towards behavioural normalisation toward mutual respect” – duplication?

**Response**: Thank you for picking this up. The sentence was removed since it repeated the content of lines 439-442.

We appreciate your valuable time and thoughtful feedback in reviewing this manuscript. We hope the revisions align with your expectations and adequately address your concerns.

Regards,
Camille Chan

On behalf of all co-authors
